# Taxonomic Revision of *Pinus fujiii* (Yasui) Miki (Pinaceae) and Its Implications for the Phytogeography of the Section *Trifoliae* in East Asia

**DOI:** 10.1371/journal.pone.0143512

**Published:** 2015-12-16

**Authors:** Toshihiro Yamada, Mariko Yamada, Minoru Tsukagoshi

**Affiliations:** 1 School of Natural System, College of Science and Engineering, Kanazawa University, Kanazawa, Japan; 2 Osaka Museum of Natural History, Higashi-sumiyoshi, Osaka, Japan; Institute of Botany, CHINA

## Abstract

*Pinus trifolia* Miki 1939 (Pinaceae) was originally proposed based on seed cones from the upper Miocene of Aichi and Gifu Prefectures, central Japan. However, before the publication of *P*. *trifolia*, a different name (*Pinus fujiii* (Yasui) Miki) was given to a female cone with the same morphology. On the other hand, *P*. *fujiii* auct. non (Yasui) Miki has been used for seed cones with different morphologies from Yasui’s holotype, i.e., apophyses arranged in 5:8 parastichies and a perexcentromucronate slightly-pointed umbo. As a result of re-examination on the Miki and Yasui specimens, we concluded that *P*. *trifolia* was a synonym for *P*. *fujiii* and proposed here *Pinus mikii* sp. nov. for cones assigned to *P*. *fujiii* auct. non (Yasui) Miki. We also emended the diagnosis of *P*. *fujiii* based on these specimens. *Pinus fujiii* is characterized by a large female cone in which the apophyses with a centromucronate prickle-like umbo are arranged in 8:13 parastichies, and deciduous seed wings. These characters suggest that *P*. *fujiii* belongs to the section *Trifoliae* of the subgenus *Pinus*, which is now restricted to North and Central America and the Caribbean islands. Fossil data suggest that the *P*. *fujiii* lineage firstly appeared in Japan around the Eocene/Oligocene boundary. We speculate that the *P*. *fujiii* lineage might have moved southward to Japan from a refugium located elsewhere in high-latitude areas in response to the late Eocene cooling event, as occurred with other *Trifoliae* species in North America.

## Introduction

Extant species of the section *Trifoliae* Duhamel [1] (subgenus *Pinus*, genus *Pinus* L. [2], Pinaceae Spreng. ex F.Rudolphi [3]) are placed into three subsections [4], *Contortae* Little and Critchfield [5], *Australes* Loudon [6], and *Ponderosae* Loudon [7], and are restricted in their distribution to North and Central America and the Caribbean islands [4, 7–9]. The *Trifoliae* are characterized by two to five leaves clustered in a persistent fascicle sheath and deciduous (articulated) seed wings, although there are a few exceptional species [4, 10]. In most species of this section, the female cones have robust woody cone scales [8, 11].

Fossil records of the *Trifoliae* in the Eocene and thereafter are centered on North America [12–21], while only a few fossils from the Miocene to Pliocene in Europe have been placed as members of the section [10, 22, 23]. Therefore, it is inferred that the *Trifoliae* originated and diversified in North America [15, 24]. This inference is also supported by phytogeographic history reconstructed from molecular phylogeny [4, 25]. However, fossil records and the molecular clock suggest different ages for the divergence of the *Trifoliae*, i.e., the Late Cretaceous (95 Ma [25]) or the early Miocene (18 Ma [9, 26]) for the molecular clock vs. the Eocene (45–50 Ma) for fossil records [15].

*Pinus trifolia* Miki is a species represented in the early late Miocene flora of Japan [27–29]. It was originally instituted based on material from the Tokiguchi Formation in Mizunami-shi, Gifu Prefecture (Pref.), and the Seto Formation in Seto-shi, Aichi Pref. [30]. This species is characterized by a large female cone with thick cone scales arranged in 8:13 parastichies, detachment of the basal female cone scales, uncinate apophyses with a prickle-like umbo, and three to five leaves clustered in a fascicle sheath [30]. Based on these features, Miki [30] inferred that *P*. *trifolia* is a close relative of extant *P*. *sabiniana* Dougl. of the section *Taeda* (*sensu* Pilger [31]), which is included in the subsection *Ponderosae* of the section *Trifoliae* in the most recent and prevalent classification of the genus *Pinus* [4].

If the above phylogenetic inference [30] is correct, *P*. *trifolia* provides significant evidence of a historic floristic tie between East Asia and North America. Since Japan became an archipelago by the opening of the Japan Sea between the late Oligocene and the earliest early Miocene [32], the migration of *P*. *trifolia* ancestors to Japan should have been completed before the formation of this geographical barrier. Therefore, *P*. *trifolia* would suggest that a Miocene origin of the *Trifoliae* is unlikely, if North America is the cradle of this section. However, in addition to the key characters listed above, character states should be further clarified so as to support that *P*. *trifolia* belongs to the *Trifoliae*, because some species of the subsection *Pinaster* Loudon [6] (section *Pinus*) also have some of these characters [4, 10]. Information on the position of a mucro on umbos and the mode of seed wing attachment would be useful to distinguish the *Trifoliae* from the *Pinaster* [4, 10, 33]; however, these characters are not available in Miki’s descriptions [30].

In addition to the problem on its affinity, we recently realized that *Pinus trifolia* may have a nomenclatural problem. Before the publication of *P*. *trifolia* by Miki [30], a new name, *Pinites fujiii*, was given to a female cone collected from the Seto Formation [34]. Yasui [34] stated the diagnosis for *Pinites fujiii* as “The phyllotaxy of the scales is 8/21. The end of the scale is generally wedge-shaped with the point drawn out into a hook. In the middle part of the cone the hook is elongated and deflected, while at the base it points downward.” Therefore, *Pinites fujiii* and *Pinus trifolia* apparently largely share the same female cone characters; however, the relationship between the two species has not been discussed until now.

In this study, we reexamined Yasui’s [34] and Miki’s [30] specimens, as well as other *P*. *trifolia* specimens collected by Prof. S. Miki. We propose that *Pinus trifolia* is a synonym for *Pinites fujiii* and discuss the affinity of this species to extant sections of the genus *Pinus*.

## Material and Methods

### Fossil material

All specimens used in this study (S1 Table) were borrowed from the Herbarium of the University of Tokyo (TI) and the Fossil collections of the Osaka Museum of Natural History (OSA; for details on these herbaria including contact information, see Index Herbariorum [35]). No other specimens were used in this study. Specimens were photographed using a D200 camera (Nikon, Tokyo, Japan) with an AF MICRO NIKKOR 60 mm lens (Nikon) under fluorescent illumination.

**Fig 1:**
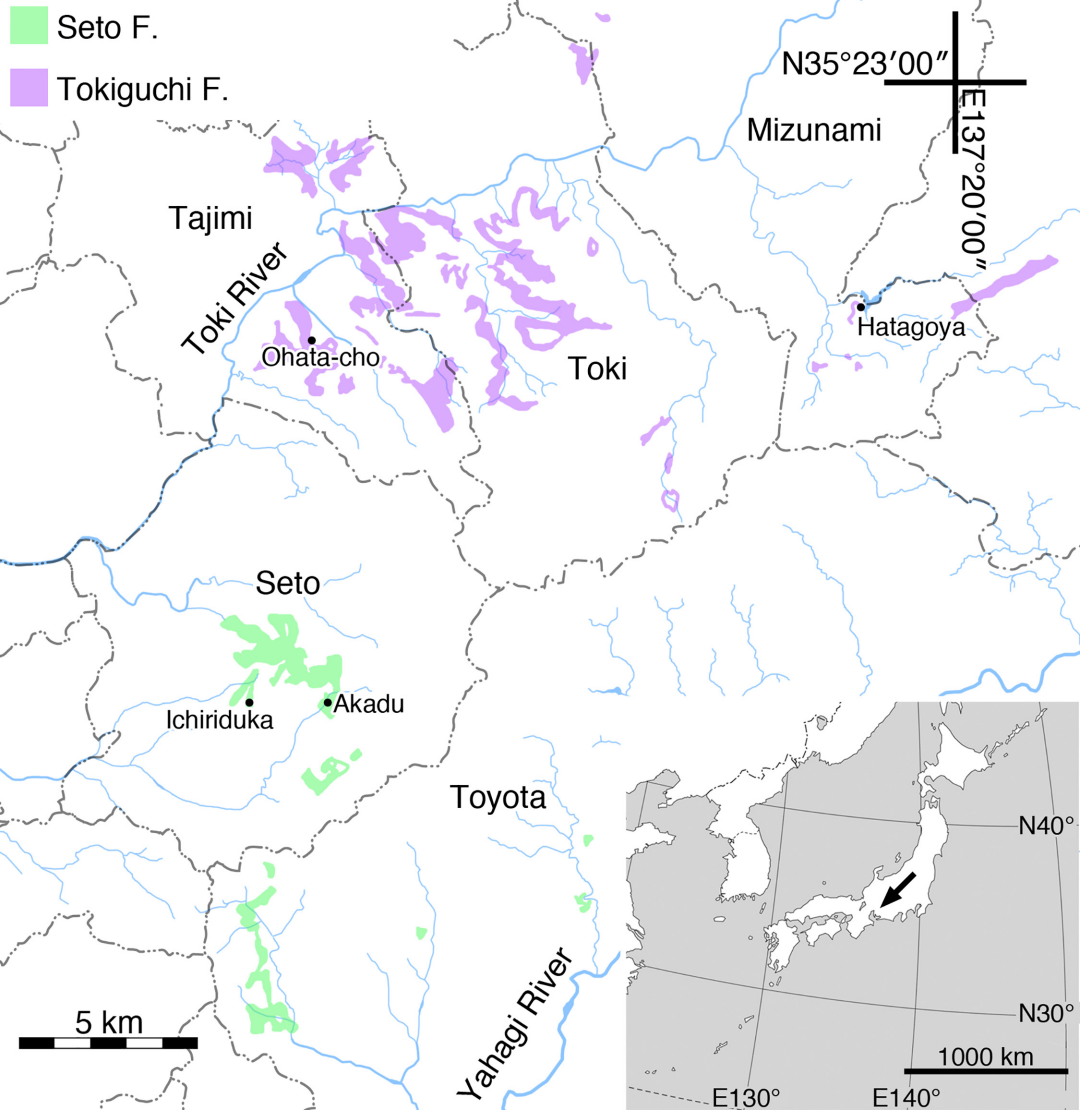
Localities of examined specimens and distributions of Seto and Tokiguchi Formations. Distributions of these formations are projected onto topographic map released by Geospatial Information Authority of Japan, based on Nakayama et al. [36].

The holotype of *Pinites fujiii* stored in TI consists of a female cone, a replica of the cone, and four microscope slides mounting sectioned parts of the cone. No specimen number is assigned to the holotype, while it is registered as “holotype of *Pinites fujiii*”. The holotype was collected in Seto-shi, Aichi Pref., Japan (Fig 1), from the Seto Formation, but the exact locality is not available.

Other specimens, including Miki’s [30] specimens, are stored in OSA F. These specimens were slightly compressed mummifications collected from the Seto or Tokiguchi Formations (Fig 1).

### Geological setting

The Seto and Tokiguchi Formations are fluvial deposits consisting of lignites, claystones, siltstones, sandstones and conglomerates [36]. These two formations were formed almost coevally in different basins close to each other (Fig 1) [36]. Their age is estimated as the early late Miocene (10.5 ± 0.4–9.7 ± 0.4 Ma), based on fission–track dating of intercalated tuff layers [37, 38].

83 plant species were reported from these two formations such as *Glyptostrobus pensilis* Koch, *Metasequoia disticha* (Heer) Miki, *Fortunearia sinensis* Rehder et E. H. Wilson, *Liquidamber formosana* Hance, *Fagus stuxbergii* (Nathorst) Tanai, *Carya striata* Miki, *Nyssa sylvatica* Marshall [27, 38]. Among them, 40 species are extinct from present Japan and extant species of the 9 genera are confined to China and North America. This composition suggests floristic ties between Japan and these regions before the early late Miocene [27].

The holotype was collected from a lignite bed consisting mainly of wood and bark fragments [34]. Although we could not know from what facies Miki’s [30] specimens were collected, it is reported that “*P*. *trifolia*” cones densely occur in semiautochthonous plant litters which contained in fine- to coarse-grained sandstone beds of channel bar deposits [38]. Miki [30] collected other organs of genus *Pinus* along with cones from the same horizons, supporting that these *Pinus* remains were not transported for a long distance from their living sites.

### Phylogenetic analyses

Phylogenetic analyses were conducted based on the morphological character matrix of Gernandt et al. [4] with some modifications. The number of vascular bundles (character #1) and resin duct position in leaves (charcter #4) were removed from the matrix because these are not available for *P*. *fujiii*. Distribution (character #9) was also removed from the matrix. Dissection of the basal cone scales was added to the matrix in which character states were coded as present (1) or absent (0) after Klaus [10] and Farjon and Styles [11].

A batch file for parsimony ratchet analysis was generated by PRAP2 [39] with settings of 1000 ratchet replicates, weight 2 and 25% weighted. Parsimony ratchet analysis was conducted by PAUP* version 4.0b10 [40] based on the batch file. Bootstrap supports were calculated by making 1000 replicates, with 10 trees held for each of 100 multiple tree-bisection-reconnection (TBR) search replications. In both parsimony ratchet and bootstrap analyses, molecular phylogeny [4] was used as a backbone constraint.

The character matrix and constraint tree used for these analyses were available as S1 and S2 Datasets.

### Nomenclature

The electronic version of this article in Portable Document Format (PDF) in a work with an ISSN or ISBN will represent a published work according to the International Code of Nomenclature for algae, fungi, and plants, and hence the new names contained in the electronic publication of a PLOS ONE article are effectively published under that Code from the electronic edition alone, so there is no longer any need to provide printed copies.

The online version of this work is archived and available from the following digital repositories: PubMed Central, LOCKSS.

## Results

### Systematic paleobotany

Order Pinales Gorozhankin, 1904 [41]

Family Pinaceae Spreng. ex F.Rudolphi, 1830 [3]

Genus *Pinus* L., 1753 [2]

### *Pinus fujiii* (Yasui) Miki emend. T. Yamada, M. Yamada et Tsukagoshi emend. nov. (Figs 2–5 herein)

**Fig 2:**
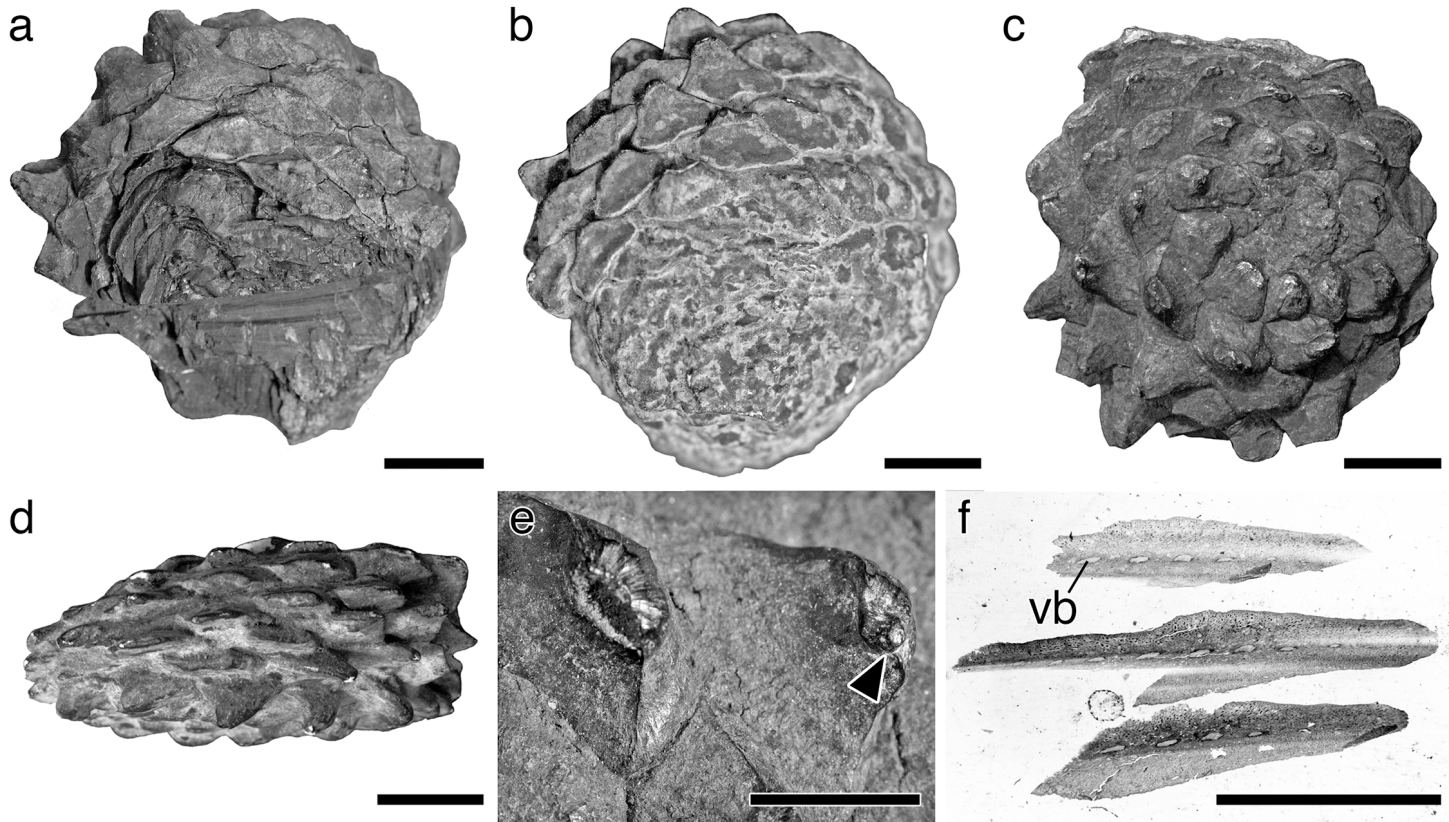
Holotype of *Pinus fujiii* and related specimen stored in TI. (a) Apical view of holotype. (b) Apical view of gypsum model casted from holotype. (c) Basal view of holotype. (d) Lateral view of gypsum model. (e) Close-up of umbos in holotype. (f) Cross section of a cone scale detached from holotype. *Arrow head* = mucro. Scale bars: a–d, 1 cm; e, f, 5 mm.

**Fig 3:**
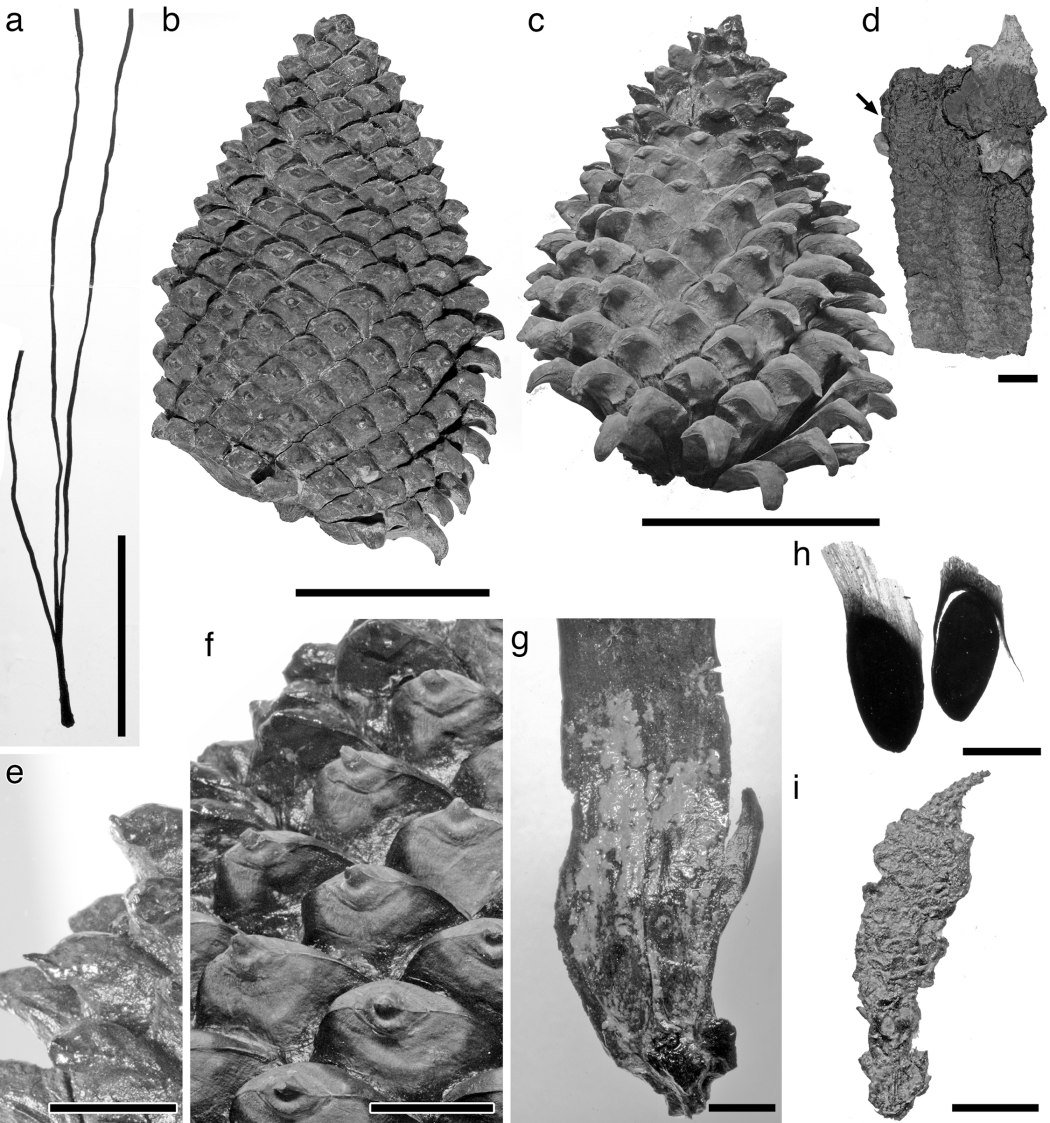
*Pinus fujiii* stored in OSA F. (a) Leaves in a sheath (OSA F4062). (b) Epitype (OSA F19363). (c) Female cone (OSA F19349–9). (d) Two female cone bases remained on branch (OSA F17476). Cone base behind branch is indicated by arrow. (e, f) Close-up of apophyses in OSA F19349–7 (e) and –8 (f). (g) Cone scale (OSA F19349–1). Seed scars are visible but seeds shown in Miki [30] are missing. (h) Seeds (2905). Note detached wing in right one. i. Male cone (OSA F19351–3). Scale bars: a–c, 5 cm; d–f, 1 cm; g–h, 5 mm.

**Fig 4:**
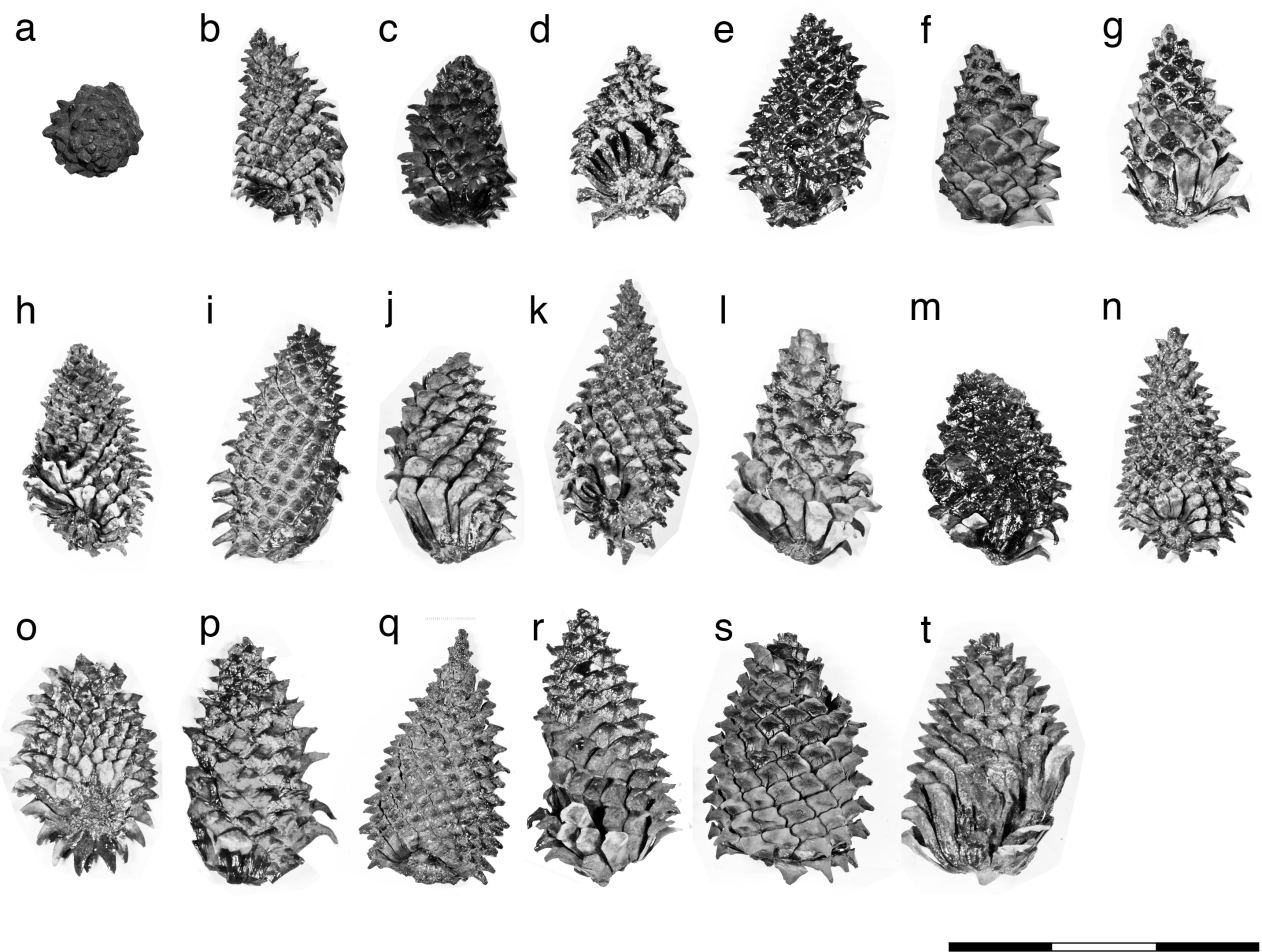
Female cones of Akadu population. **(**a) Holotype. (b) OSA F19355–3. (c) OSA F19355–1. (d) OSA F19356–2. (e) OSA F19356–5. (f) OSA F19355–2. (g) OSA F19356–1. (h) OSA F19355–5. (i) OSA F19356–7. (j) OSA F19356–4. (k) OSA F19355–7. (l) OSA F19356–6. (m) OSA F19355–4. (n) OSA F19355–6. (o) OSA F19356–3. (p) OSA F19356–10. (q) OSA F19355–8. (r) OSA F19356–9. (s) OSA F19355–9. (t) OSA F19356–8. Scale bar: 15 cm.

**Fig 5:**
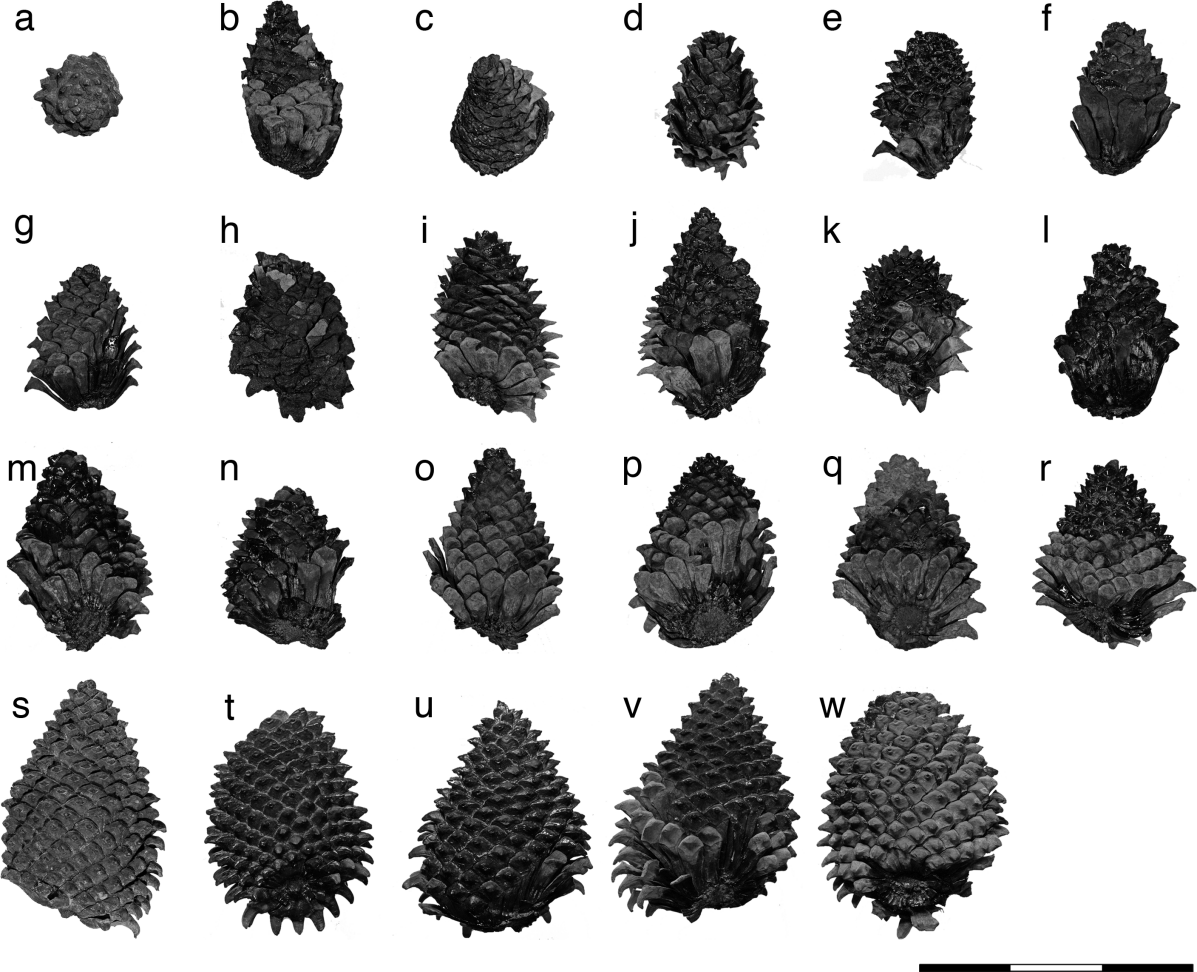
Female cones of Hatagoya population. (a) Holotype. (b) OSA F19296–10 (c) OSA F19296–1 (d) OSA F19296–4. (e) OSA F19296–2. (f) OSA F19296–5. (g) OSA F19296–3. (h) OSA F19296–7. (i) OSA F19296–11. (j) F19296–17. (k) OSA F19296–6. (l) OSA F19296–9. (m) OSA F19296–16. (n) OSA F19296–8. (o) OSA F19296–15. (p) OSA F19296–14. (q) OSA F19296–13. (r) OSA F19296–12. (s) Epitype (OSA F19363). (t) OSA F19349–11. (u) OSA F19349–9. (v) OSA F19349–10. (w) OSA F19349–8. Scale bar: 15 cm.

*Pinus fujiii* (Yasui) Miki, Miki,1939: p. 245 (nomenclatural note only for new combination) [30]

#### Basionym

*Pinites fujiii* Yasui, Yaui, 1928: p. 437, Text-figure 12, Figures 83–-85 in Plates 20, 21 [34]

#### Synonym

*Pinus trifolia* Miki, Miki, 1939: p. 244, Text-figure 3, Plate 4 [30]; Miki 1948: p. 255 (with no illustration) [23]; Miki, 1957: p. 253, Text-figures 7A, 8Cc, Plate 6 [42]; Tanai, 1961: p. 256, Figure 8 in Plate 2 [29]; Omori and Tanaka, 1965: p. 567, Figure 2 [43]; Tsukagohi and Todo Collaborative Research Group, 1998: p. 496, Figures 3.1, 3.2 [44]; Ando et al., 1999: p. 19, Figures 1, 4 [38]; Nakayama et al., 1999: p. 3, Figures 1, 2 [45]; Nirei and Akiyama, 2011: p. 102, Figure 4 [46]

#### Holotype

Yasui’s female cone [34] (Figs 2, 4A and 5A herein). The holotype is stored in TI, but no specimen number has been assigned since1928.

#### Type strata

The upper Miocene Seto Formation, Tokai Group

#### Type locality

Seto-shi, Aichi Pref., Japan (exact locality unknown)

#### Epitype

OSA F19363 (Designated here [Figs 3B and 5S]; collected from Hatagoya, Mizunami-shi, Gifu Pref., Japan; firstly illustrated by Miki [30] in Text-figure 3C and Figure E of Plate 4)

#### Other specimens examined

OSA F2905 (Fig 3H), OSA F2908, OSA F2909, OSA F17476 (Fig 3D), OSA F19296–1 (Fig 5C), –2 (Fig 5E), –3 (Fig 5G), –4 (Fig 5D), –5 (Fig 5F), –6 (Fig 5K), –7 (Fig 5H), –8 (Fig 5N), –9 (Fig 5L), –10 (Fig 5B), –11 (Fig 5I), –12 (Fig 5R), –13 (Fig 5Q), –14 (Fig 5P), –15 (Fig 5O), –16 (Fig 5M), –17 (Fig 5J), OSA F19351–1 (Miki, 1939: 2nd right of Figure B in Plate IV [30]), –2, –3 (Fig 3I herein; Miki, 1939: 2nd left of Figure B in Plate IV [30]), OSA F19349–1 (Fig 3G herein; Miki, 1939: Text-figure 3E, Figure D in Plate IV [30]), –2 (Miki, 1939: Text-figure 3I, bottom of Figure C in Plate IV [30]), –3 (Miki, 1939: top of Figure C in Plate IV [30]), –4 (Miki, 1939: Text-figure 3Da, center of Figure H in Plate IV [30]), –5 (Miki, 1939: Text-figures 3D–b,–c, left of Figure H in Plate IV [30]), –6 (Miki, 1939: right of Figure H in Plate IV [30]), –7 (Fig 3E herein; Miki, 1939: Text-figure 3B, Figure G in Plate IV [30]), –8 (Figs 3F and 5W herein; Miki, 1939: Figure F in Plate IV [30]), –9 (Figs 3C and 5U), –10 (Fig 5V), –11 (Fig 5T), OSA F19355–1 (Fig 4C), –2 (Fig 4F), –3 (Fig 4B), –4 (Fig 4M), –5 (Fig 4H), –6 (Fig 4N), –7 (Fig 4K), –8 (Fig 4Q), –9 (Fig 4S), OSA F19356–1 (Fig 4G), –2 (Fig 4D), –3 (Fig 4O), –4 (Fig 4J), –5 (Fig 4E), –6 (Fig 4L), –7 (Fig 4I), –8 (Fig 4T), –9 (Fig 4R), –10 (Fig 4P). For details, see S1 Table.

#### Emended Diagnosis

Female cone ovoid or oblong ovoid with asymmetrical base; cone scales in 8:13 parastichies; apophysis thick, pentagonal, bend downwardly on the abaxial side of the cone; umbo somewhat flattened, centromucronate; mucro forming distinct prickle; vascular bundles in cone scale up to 11, arranged in one plane.

#### Description

**Fig 6:**
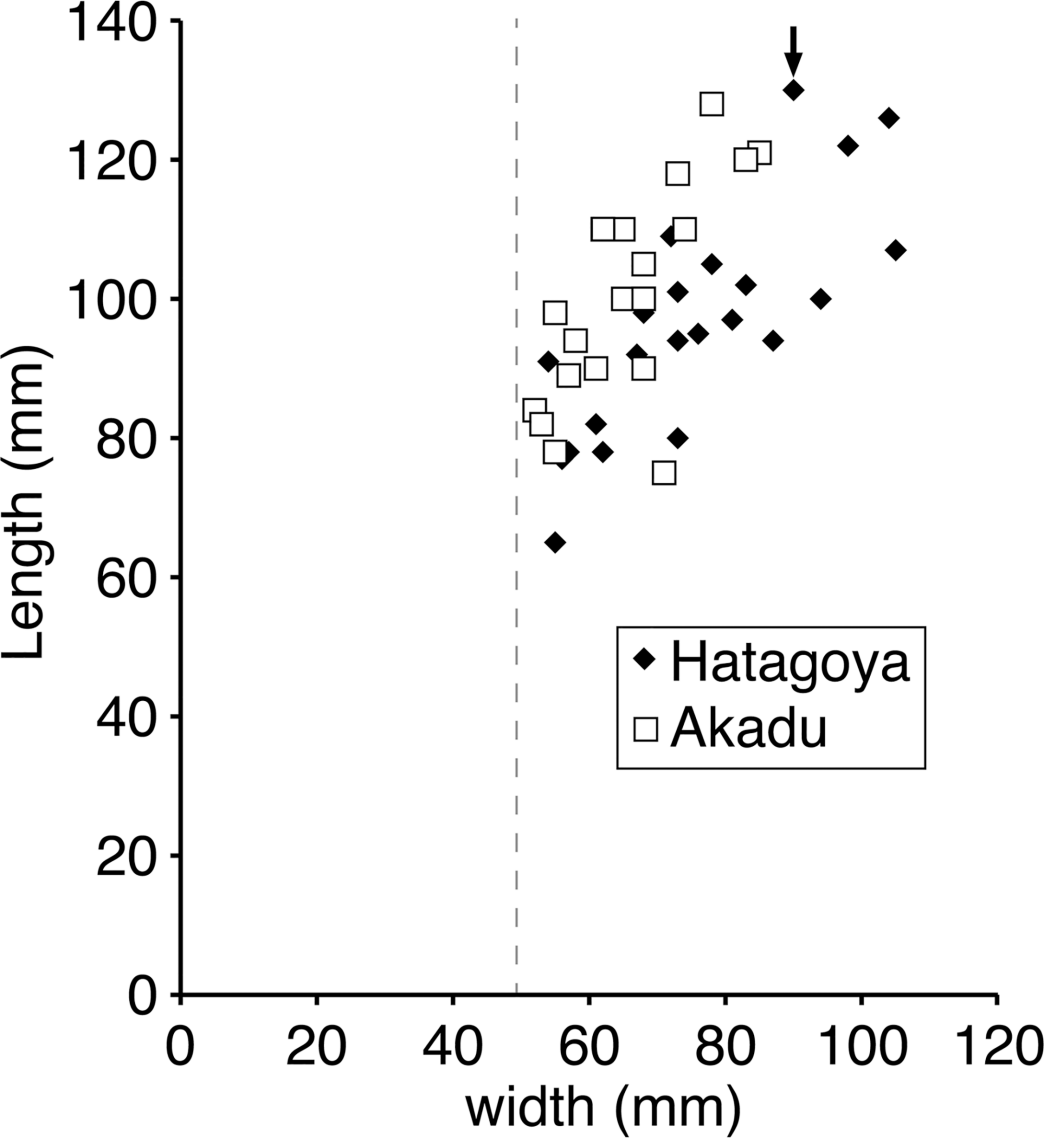
Size variations of female cones found in Akadu and Hatagoya populations. Width of holotype and size of epitype are indicated by dashed line and arrow, respectively.

The female cone is ovoid or oblong ovoid with an asymmetrical base, 6.5–13 cm long, and 4.9–10 cm wide when it is fully grown (Figs 2, 3B and 3C and Figs 4–6). The basalmost cone scales usually remain on the branch when the cone has fallen off (Fig 3D). Cone scales are arranged in 8:13 parastichies. Up to 11 vascular bundles are coplanarly arranged in the middle part of a cone scale (Fig 2F. see also [34]). Apophyses are thick, 10–13 mm high, 15–20 mm wide, and pyramidally risen (Figs 2D, 3B and 3C). The risen part of an apophysis is rolled downward on the abaxial (away from the branch) side of a cone, but the bend is not as distinct on the adaxial side (Figs 2D, 3B and 3C). The umbo is centromucronate and the mucro forms a distinct prickle (Figs 2E, 3E and 3F). The seed is articulated to the wing, elliptic, 9–10 mm long, and 4–5 mm wide (Fig 3H). The seed wing is 13–16 mm long and 8 mm wide (Fig 3G. See also [30]).

#### Nomenclatural note

**Fig 7:**
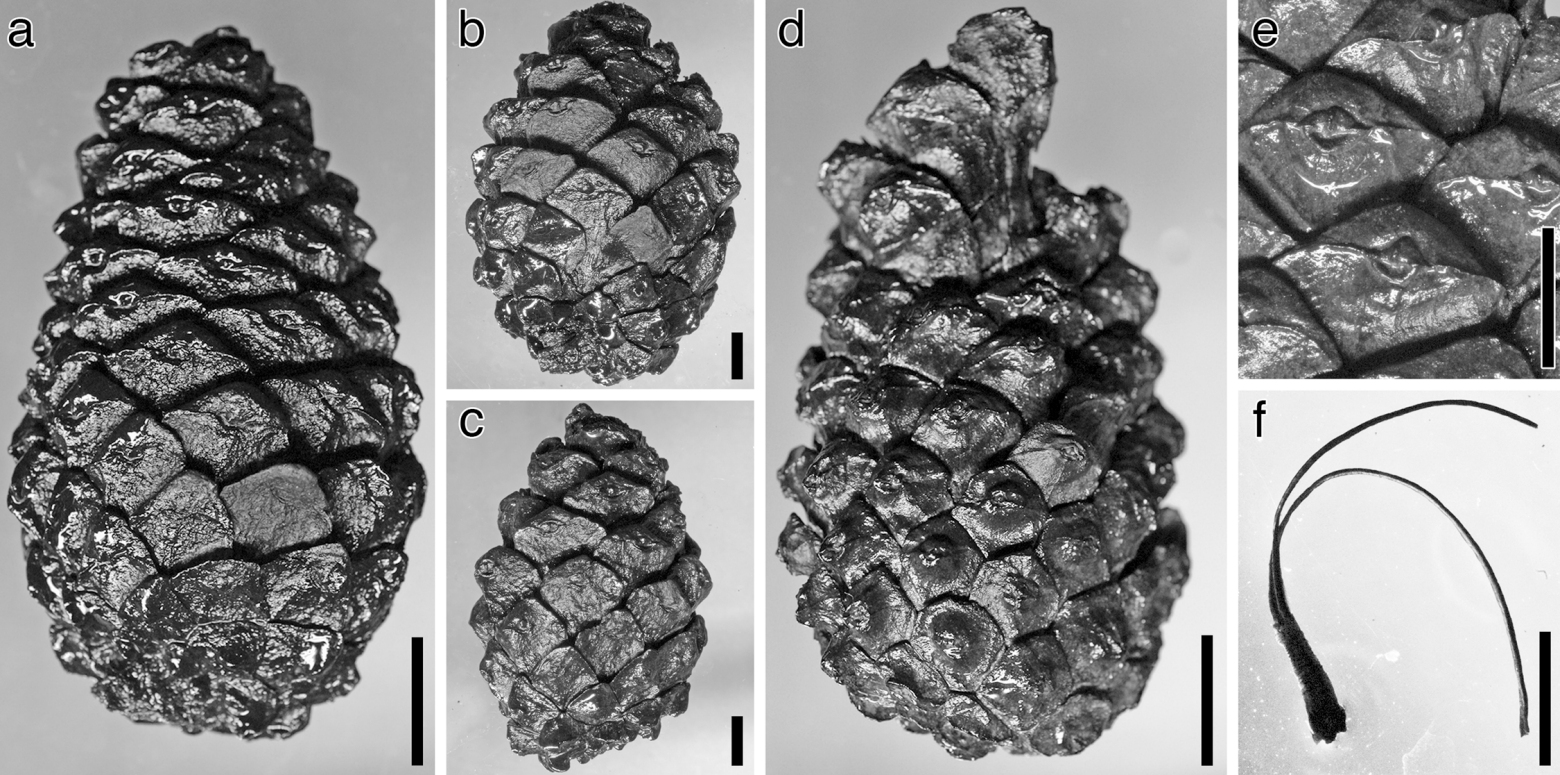
*Pinus mikii* sp. nov. stored in OSA F. (a) Holotype (20241–1). (b, c) Paratypes (20241–2, –3, respectively) (d) Female cone (19432–1). (e) Close-up of apophyses (19432–2). f. Leaves (20242–1). Scale bars: a, d, 1 cm; b, c, e, f, 5 mm.

In 1939, Miki [30] legitimately proposed a new combination *Pinus fujiii* for *Pinites fujiii* based on Yasui’s holotype [34] since genus *Pinites* Lindley and Hutton 1832 was originally given for Carboniferous woods [47] which have possible affinity with the Araucariaceae [48]. Later, he reported some female cones (Fig 7A–7C) and leaves (Fig 7F) from the Tokiguchi Formation and assigned them to “*Pinus fujiii* (Yasui) Miki” [27]. Since then, many authors have identified “*P*. *fujiii*” based on this report [27], rather than the original description by Yasui [34]. However, Yasui’s holotype of *P*. *fujiii* [34] is clearly different from the female cones reported by Miki [27]. Therefore, we establish a new species below for “*Pinus fujiii*” *sensu* Miki 1941 [27], based on his specimens.

#### Comparison

Ten fossil species of subgenus *Pinus* are known that have large female cones (greater than 10 cm long) with centromucronate umbos (Table 1). Among them, *P*. *fujiii* is similar in female cone characteristics to *P*. *engelhardtii* (Menzel) Mai from the lower Miocene of the Czech Republic [22, 23], *P*. *lawsoniana* from the middle Pliocene of California (CA), U.S.A. [14], *P*. *piperi* Dorf from the upper Miocene to Pliocene of CA [18, 49], and *P*. *truckeensis* Axelrod from the upper Miocene of Nevada, U.S.A. [15], i.e., ratios of width to length are greater than 0.5 and they exhibit pyramidally-risen apophyses. However, the bending of the risen part in the abaxial apophyses, a characteristic of *P*. *fujiii*, is not distinct in the former three species. *Pinus engelhardtii* is also different from *P*. *fujiii* in the indistinct mucro [22, 23]. *Pinus truckeensis* has the most similar appearance to *P*. *fujiii* among the four species, but apophyses of *P*. *truckeensis* are larger than those of *P*. *fujiii* [15]. In addition, apophyses have a greater width than height in *P*. *truckeensis* [15], while the width is almost the same as the height in apophyses of *P*. *fujiii*.

**Table 1:** Comparison between *P*. *fujiii* and other species characterized by a large female cone with centromucronate umbos.

Species	Cone length (L, cm)	Cone width (W, cm)	Ratio (W/L)	Uncinate apophyses	Age	References
*P*. *fujiii*	6.5–13	4.9–10	0.56–0.98	present	late Miocene	[30, 34]
*P*. *celetomensis*	14–15	7.5	0.5–0.54	absent	late Miocene	[15]
*P*. *diegensis*	9.0–11	5.0–6.5	0.56–0.59	absent	late Pliocene	[16]
*P*. *engelhardtii*	4.6–13	2.1–7.5	0.46–0.63	absent	early Miocene	[22, 23]
*P*. *engelmannoides*	11–12	4.5	0.38–0.41	absent	late Oligocene	[15]
*P*. *lawsoniana*	8.0–10.5	7.0	0.67–0.88	absent	middle Pliocene	[14]
*P*. *piperi*	10.2	12.5	1.2	absent	late Miocene–Pliocene	[18, 49]
*P*. *riogrande*	12–15 <	4.5–6.0	< 0.4	?	late Oligocene	[15]
*P*. *spinosa*	14	3.0–4.0	0.21–0.29	absent	Pliocene	[22]
*P*. *stroboides*	10 <	ca. 3.0	< 0.3	absent	late Eocene	[22]
*P*. *truckeensis*	17–18	11	0.61–0.65	present	late Miocene	[15]

There are two records of “*Pinus trifolia* Miki” from the late Eocene to early Oligocene in Japan: cones from an unknown locality of Kyushu [50] and leaves, female cones, and male cones from the Kobe Group (37–31 Ma [51]) distributed in Kobe-shi, Hyogo Pref. [52]. Comparison between *P*. *fujiii* and the cones from Kyushu is difficult because these cones are poorly preserved. It is suggested that specimens from the Kobe Group would belong to a closely related but distinct species from *P*. *fujiii* because the male cones are twice the size of those of *P*. *fujiii* [52].

#### Remarks

Along with female cones, some detached male cones and leaves were discovered from the same horizons [30] (For details on the specimens, see S1 Table). Three or four, rarely five, leaves are clustered in a persistent fascicle. The leaf sheath is 10–12 mm long (Fig 3A). The male cone has a woody axis, is stalked, 15–25 mm long and 5–6 mm wide (Fig 3I).

### *Pinus mikii* T. Yamada, M. Yamada et Tsukagoshi sp. nov. (Fig 7 herein)

#### Synonym

*Pinus fujiii* auct. non (Yasui) Miki, Miki, 1941: p. 255, Text-figures 5K–L, Figure G in Plate 4 [27]; Miki, 1957: p. 250, Text-figures H–K, Plate 7 [42]; Tanai, 1961: p. 255, Figure 9 in Plate 2, *non* Figure 10 in Plate 3 [29]; Kimura et al., 1981: p. 91, Text-figures 2a–c, Figures 2–3 in Plate 9 [53]; Ando et al., 1999: Figure 5.1 [38]; Nakayama et al., 1999: Figure 6.1 [45]; Saneyoshi et al., 2000: Figure 6–1 [37]; Sawada et al., 2013: Figure 5 [54]; Yamada et al., 2014: p. 200, Figures 3a–f [55]; Yamada and Yamada, 2014: p. 29, Figures 1A–E [56]; *Pinus miocenica* auct. non Tanai, Matsuo, 1963: Figure 5 in Plate 43 [57]; Ina, 1981: Figure 1 in Plate 2 [58]

#### Holotype

OSA F20241–1 (Designated here [Fig 7A]; Miki,1941: the left specimen of Text-figure 5L [27])

#### Paratype

OSA F20241–2 (Designated here [Fig 7B]; Miki, 1941: the right specimen of Text-figure 5L [27]), –3 (Designated here [Fig 7C]; Miki, 1941: the center specimen of Text-figure 5L [27])

#### Other specimens examined

OSA F19432–1 (Fig 7D; Miki, 1957: Figure Hb in plate 7 [42]), OSA F19432–2 (Fig 7E)

#### Type strata

The upper Miocene Tokiguchi Formation, Tokai Group

#### Type locality

Ohata-cho 3-chome (formerly called as ‘Ichinokuraguchi’), Tajimi-shi, Gifu Pref., Japan (Fig 1).

#### Etymology

Commemorating late Prof. Shigeru Miki who conducted the first comprehensive study on the Japanese fossil Pinaceae and greatly contributed to the clarification of the Neogene vegetation of Japan.

#### Diagnosis

Female cones conical to ovoid, with a cordate base; cone scales in 5:8 parastichies; apophyses rhombic, moderately swollen, with transverse keel and radiating ridges, vallate; umbos present at the upper one-third to one-half of dorsal apophyses, weakly depressed on basal half, perexcentromucronate; mucros slightly hooked, prominent even in basal cone scales.

#### Description

Female cones are long elliptic to oblong ovoid, 40–70 mm long, and 18–37 mm wide (Fig 7A–7D). The cone base is cordate with a short peduncle (Fig 7A–7C). Cone scales are arranged in 5:8 parastichies (Fig 7A–7D). The apophysis is rhombohedral to hexagonal, moderately swollen, 8–12 mm wide, and 5–7 mm high at the middle of the cone (Fig 7A–7E). A weak transverse keel and radiating ridges are developed on the apohyses (Fig 7E). The umbo is rhombic to elliptic, 1.6–2.6 mm wide by 1.5–2 mm high, and located one-half to one-third from the upper corner of the apophysis (Fig 7E). The lower half of the umbo is depressed (Fig 7E). An obtusely pointed mucro develops at the upper-center (perexcentromucronate) of the umbo (Fig 7E).

#### Remarks

Some leaves occur in association with female cones of *P*. *mikii* described above (For details, see S1 Table). Leaves are linear, in bundles of two, and up to 1.5 mm wide. A single ridge is present on the abaxial side. The leaf sheath is persistent (Fig 7F).

Yamada et al. [55] compared *P*. *fujiii* auct. non (Yasui) Miki (*P*. *mikii* in this study) to other fossil and extant species. We inferred that this species has an affinity with extant *P*. *thunbergii* Parl. mainly distributed in Japan, as well as *P*, *hwangshanensis* W.Y. Hsia and *P*. *tabuliformis* Carrière which are distributed in China [55]. This inference suggests phytogeographyic tie between Japan and China during the Miocene [29].

## Discussions

### *Pinus trifolia* Miki is a synonym for *Pinites fujiii* Yasui

In 1928, *Pinites fujiii* was established as a new species based on a single female cone from the upper Miocene Seto Formation reported by Yasui [34]. This species is characterized by cone scales arranged in 8:13 parastichies (Fig 1C), apophyses bending downwardly on one side of the cone (Fig 1C and 1D), and umbos with a centrally positioned prickle-like mucro (Fig 2E) [34]. Later, in 1939, a new combination *Pinus fujiii* was legitimately proposed for *Pinites fujiii* based on Yasui’s [34] holotype [30]. At the same time, *Pinus trifolia* was established as a new species by Miki, based on female and male cones, leaves, pollen, and seeds from the Tokiguchi and Seto Formations (Fig 3) [30]. However, Miki’s [30] female cones of *P*. *trifolia* (Fig 3B, 3C, 3E and 3F) clearly exhibit the diagnostic characteristics of the *Pinites fujiii* specimen reported by Yasui [34].

Although we do not know the exact length in Yasui’s holotype because it is compressed vertically [34], it is half the size of Miki’s female cones of *P*. *trifolia* [30], judging from the width. To evaluate whether *P*. *trifolia* differs from *P*. *fujiii* in the size of the female cone, we examined variations in female cone sizes within a population by using specimens collected from Akadu or Hatagoya. The size of cones varied continuously in both localities, i.e., 52–85 mm wide and 75–128 long in Akadu (Figs 4 and 6), and 54–105 mm wide and 65–130 long in Hatagoya (Figs 5 and 6), and the minimum width in each population was slightly wider than that of Yasui’s holotype [34] (49 mm; Figs 1 and 4–6). Therefore, it is reasonable to conclude that Yasui’s holotype [34] and Miki’s cones [30] could be small and large cones of a single species, respectively.

Yasui’s holotype [34] would be an immature cone that was not spontaneously detached from a branch. In *P*. *fujiii*, the basal cone scales remained on the branch (Fig 3D) when the cones were excised, as seen in Miki’s female cones [30] (Figs 3B, 3C, 4 and 5). However, such excision is not observed in the holotype (Fig 2C). The holotype was found in a lignite bed mainly composed of large branches, wood, and bark remains [34], while the other specimens used in this study were collected from clay or sandy siltstone beds. The holotype might have been trapped in the sediment along with the branch bearing it, while excised cones were transported to the depositional place in a different manner from the stunted cone.

In conclusion, *Pinus trifolia* Miki is a synonym for *Pinus fujiii* (Yasui) Miki. However, Yasui’s diagnosis [34] was solely based on a putatively immature female cone. Thus, we propose here an emended specific diagnosis by integrating diagnostic features stated in both studies [30, 34]. It is suggested that one Miki’s [30] specimen is designated as an epitype that represents a fully-grown female cone.

### Affinity of *Pinus fujiii* to extant species

*Pinus fujiii* has leaves clustered in groups of three or four, rarely in five (Fig 3A). This character is commonly found in most extant species of the *Trifoliae* (subgenus *Pinus*), especially in species of the subsection *Ponderosae* [4, 11]. Exceptionally in the section *Pinus*, two species of the subsection *Pinaster* also have three leaves per fascicle, i.e., *P*. *canariensis* C. Sm. and *P*. *roxburghii* Sarg. [4, 59]. However, cones of *P*. *canariensis* and *P*. *roxburghii* have excentromucronate umbos [10], while cones of *P*. *fujiii* have centromucronate umbos (Fig 3D and 3E), like many *Trifoliae* species [4, 11, 15]. In addition, seeds of *P*. *canariensis* and *P*. *roxburghii* are adnate to the wing [10], contrary to the articulated seeds found in both *P*. *fujiii* (Fig 3H) and the *Trifoliae* species [4, 11].

Yasui [34] inferred that *P*. *fujiii* is closely related to *P*. *pinaster* Aiton of the subsection *Pinaster* because the vascular bundles of the cone scales are arranged in a single plane in both species (Fig 2F) [34]. However, similar arrangements are also found in some *Trifoliae* species, such as *P*. *palustris* Mill. and *P*. *rigida* Mill. (M. Yamada, unpublished data). The two leaves per sheath in *P*. *pinaster* [7, 10] are also different from the three to five leaves found in *P*.*fujiii*.

**Fig 8:**
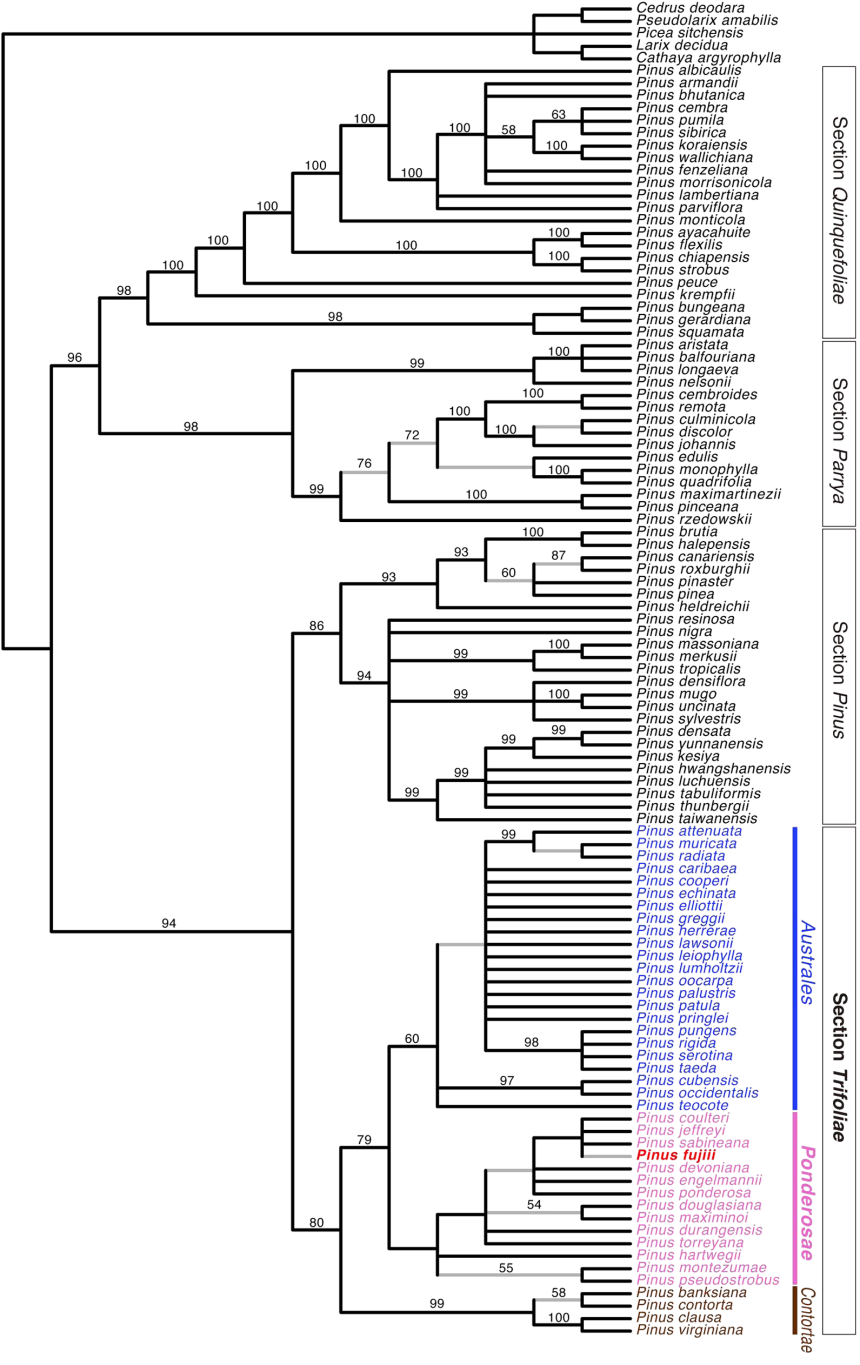
Strict consensus tree of 112 most parsimonious trees (length = 56, consistency index = 0.196, retention index = 0.804). Bootstrap values (> 50%) are shown above branches. Branches not resolved in the backbone constraint tree [4] are indicated in gray.

These morphological characters seem to support Miki’s [30] inference that “*P*. *trifolia*” is a species of the section *Taeda* (*sensu* Pilger [31]) which is now included in the section *Trifoliae* (*sensu* Gernandt et al. [4]). To test objectively if these characters support the affinity of *P*. *fujiii* with the *Trifoliae*, we conducted a phylogenetic analysis based on them by using molecular phylogeny of the extant species as a backbone constraint. As a result, *P*. *fujiii* was included in a clade consisting of extant *Australes* and *Ponderosae* species within the *Trifoliae* clade with 79% bootstrap supports (Fig 8). *Pinus coulteri* D. Don, *P*. *fujiii* and *P*. *jeffreyi* Balf. and *P*. *sabineana* formed a clade within the *Ponderosae* clade, but support for this clade was not sufficient (Fig 8). Since small number of characters was used for this analysis, the obtained result is a preliminary one. However, the result clearly shows that Miki’s [30] inference is reasonable as long as the morphological characters available for *P*. *fujiii* are concerned.

Some molecular clock-based studies suggested that the *Trifoliae* was originated in North America in the early Miocene [9, 26], contrary to the Paleogene fossil records [15, 21] and molecular dating suggesting pre-Eocene divergence [25]. If this would be the case, our phylogenetic inference implies that the ancestor of *P*. *fujiii* was dispersed from North America to Japan during the Miocene in spite of a huge geographic barrier between them [32]. Alternatively, the result of our phylogenetic analysis would be artifact despite many characters shared between *P*. *fujiii* and the extant *Trifoliae*. Phylogenetic analysis incorporating more morphological characters, as well as molecular dating based on other methods and/or markers, would be helpful to resolve this discrepancy between paleobotanical and neobotanical data.

### Implications of *Pinus fujiii* on history of pines during Cenozoic

Extant species of the section *Trifoliae* are restrictively distributed in North and Central America and the Caribbean islands [4, 7–9]. Many fossil species of this section have also been reported from the Eocene to Pleistocene in North America [12–21]; thus North America is considered to be the cradle of this section [15, 24]. In the traditional paleobotanical scenario on North American pines, the distribution of the *Trifoliae* is considered to be retracted to refugia that were located in the high and low latitudes and middle latitude uplands during the Eocene [15, 24] when the climate was globally humid and warm [60]. The *Trifoliae* expanded their distribution from the refugia around the Eocene/Oligocene boundary [15, 24] as the climate became drastically cooler and drier in this period [60].

A possible ancestor of *P*. *fujiii* [52] appeared in Japan around the late Eocene to early Oligocene (ca. 37–31 Ma [51]) when the backbone of the Japanese Archipelago was still connected to the Eurasian Continent [32]. Since *Trifoliae*-like fossils are extremely rare in eastern Eurasia, it is not clear when and where a lineage giving rise to *P*. *fujiii* first appeared there. However, it is possible to hypothesize that the *Trifoliae* retreated to the high-latitudes of North America [24] and migrated to the high-latitudes of eastern Eurasia by passing through high-latitude corridors in clockwise or counterclockwise directions during the Eocene [61, 62]. Ancestor of *P*. *fujiii* might have originated from this migrated population. Alternatively, the ancestor might have arrived directly in Japan from high-latitude refugia of North America through the Beringian Corridor around the Eocene/Oligocene boundary. Whichever is the case, the late Eocene cooling event [60] enabled the *P*. *fujiii* lineage to move southward to Japan, as was the case with other *Trifoliae* species in North America [15, 24]. The identification of Paleogene fossils with *Trifoliae*-like appearance in eastern Eurasia would help greatly in tracing the migration history of *P*. *fujiii*.

## Supporting Information

S1 Dataset Morphological character matrix used for phylogenetic analyses. (NEX)

S2 Dataset Backbone constraint tree used for phylogenetic analyses. (NEX)

S1 Table Specimens used for this study. (XLSX)
